# The Dutch Birth Centre Study: study design of a programmatic evaluation of the effect of birth centre care in the Netherlands

**DOI:** 10.1186/s12884-015-0585-1

**Published:** 2015-07-16

**Authors:** Marieke A.A. Hermus, Therese A. Wiegers, Marit F. Hitzert, Inge C. Boesveld, M. Elske van den Akker-van Marle, Henk A. Akkermans, Marc A. Bruijnzeels, Arie Franx, Johanna P. de Graaf, Marlies E.B. Rijnders, Eric A.P. Steegers, Karin M. van der Pal-de Bruin

**Affiliations:** Department of Child Health, TNO, PO Box 2215, 2301 CE Leiden, The Netherlands; Department of Obstetrics, Leiden University Medical Center, PO Box 9600, 2300 RC Leiden, The Netherlands; Midwifery Practice Trivia, Werkmansbeemd 2, 4907 EW Oosterhout, The Netherlands; NIVEL (Netherlands Institute for Health Services Research), PO Box 1568, 3500 BN Utrecht, The Netherlands; Department of Obstetrics and Gynaecology, Erasmus University Medical Centre, PO Box 2040, 3000 CA Rotterdam, The Netherlands; Jan van Es Institute, Netherlands Expert Centre Integrated Primary Care, Randstad 2145-a 1314 BG, Almere, The Netherlands; Department of Medical decision making, Leiden University Medical Center, PO Box 9600, 2300 RC Leiden, The Netherlands; Department of Management, Tilburg School of Economics and Management, PO Box 90153, 5000 LE Tilburg, The Netherlands; Division Woman and Baby, University Medical Centre Utrecht, PO box 85500, 3508 GA Utrecht, The Netherlands

**Keywords:** Birthing centres, Delivery rooms, Delivery obstetric, Pregnancy outcome, Home childbirth, Midwifery, Communication, Outcome assessment (Health care), Perinatal mortality, Integrated care

## Abstract

**Background:**

Birth centres are regarded as settings where women with uncomplicated pregnancies can give birth, assisted by a midwife and a maternity care assistant. In case of (threatening) complications referral to a maternity unit of a hospital is necessary. In the last decade up to 20 different birth centres have been instituted in the Netherlands. This increase in birth centres is attributed to various reasons such as a safe and easy accessible place of birth, organizational efficiency in integration of care and direct access to obstetric hospital care if needed, and better use of maternity care assistance. Birth centres are assumed to offer increased integration and quality of care and thus to contribute to better perinatal and maternal outcomes. So far there is no evidence for this assumption as no previous studies of birth centres have been carried out in the Netherlands.

**Design:**

The aims are 1) Identification of birth centres and measuring integration of organization and care 2) Measuring the quality of birth centre care 3) Effects of introducing a birth centre on regional quality and provision of care 4) Cost-effectiveness analysis 5) In depth longitudinal analysis of the organization and processes in birth centres.

Different qualitative and quantitative methods will be used in the different sub studies. The design is a multi-centre, multi-method study, including surveys, interviews, observations, and analysis of registration data and documents.

**Discussion:**

The results of this study will enable users of maternity care, professionals, policy makers and health care financers to make an informed choice about the kind of birth location that is appropriate for their needs and wishes.

## Background

The Dutch maternity care system is based on the notion that pregnancy, birth and the puerperium are primarily physiological processes. Most pregnant women are initially considered as ‘low risk’ and in 2012 85 % of them initially received antenatal care from an independently operating community midwife. The remaining 15 % of pregnant women received antenatal care from a secondary or tertiary obstetrician from the beginning of pregnancy onwards, mostly due to a history of medical or obstetrical problems [1]. If risk factors arise during pregnancy, during labour or in the postpartum period, a woman is referred to secondary care. Secondary care is provided under the responsibility of an obstetrician and clinical midwives or trainee obstetricians can be involved. This risk selection and role division between the professions is based on the List of Obstetric Indications, a document that designates the appropriate level of care for more than a hundred obstetrical conditions [2]. Interventions such as augmentation of labour, pharmacological pain relief, continuous foetal monitoring or instrumental birth only take place in secondary or tertiary care.

One important aim of the Dutch model is to ensure safe midwifery-led care under the responsibility of an independent community midwife for women with low risk pregnancies, regardless whether they prefer to give birth at home, in a birth centre or in a hospital. The percentage of home births in the Netherlands is high compared to other developed countries but is decreasing rapidly. In 2012, 15.7 % of all births in the Netherlands took place at home compared to 30.3 % in 2000 [1]. This may be due to a changing trend in women’s choices for the planned place of birth, shifting from home to hospital, as well as to a considerable rise in non-urgent referrals to obstetrician-led care for pain relief [3, 4].

These trends led to a substantial increase of births in obstetric hospital units. To accommodate the growing number of low-risk women who do not want to deliver at home several birth centres were established in the Netherlands with a large variation in their philosophies, characteristics and service delivery [5–7]. Studies on birth centre care in other countries than the Netherlands show that low risk women who planned birth in a birth centre experience significantly fewer interventions compared to women who planned birth in a conventional labour setting, including fewer intra partum caesarean sections, and less frequent use of obstetric analgesia and augmentation of labour [8–12]. The Birthplace study in England showed that adverse perinatal outcomes were not significantly different for low risk nulliparous women who planned birth in freestanding midwifery units and alongside midwifery units compared with planned birth in an obstetric unit. For multiparous women, birth in freestanding and alongside midwifery units significantly and substantially reduced the odds of experiencing an unplanned caesarean section, instrumental birth or episiotomy [8].

The effect of the introduction of a relatively large number of birth centres on the quality and the effectiveness of the Dutch maternity care system have not been studied up to now. The objectives of the Dutch Birth Centre Study can be summarized as follows:

1. To determine process, structure and outcome quality indicators enabling the assessment of the quality of birth and postpartum care in a birth centre, in collaboration with the various care providers and clients involved;
2. To develop a typology of birth centres based on the level of integration of organisation and care, also making use of the quality indicators mentioned;
3. To assess the effect of birth centre care in relation to the different types of birth centres in terms of optimality and adverse outcomes;
4. To study the impact of the introduction of birth centre care on the local adjacent birth and postpartum care system by comparing process indicators and perinatal and maternal outcomes before and after the introduction of a birth centre;
5. To study the cost effectiveness of birth centre care compared with usual care (home birth and birth under community midwifery led care in a hospital);
6. To assess experiences of both clients and care givers (working either within or in collaboration with a birth centre);
7. To perform a longitudinal multiple case study investigating the organizational processes in a limited number of selected birth centres from an operational, medical, behavioural and administrative perspective;
8. To translate results of this study into recommendations for future birth and postpartum care in the Netherlands;

In this paper we introduce the Dutch Birth Centre Study and its design.

## Methods/design

### Study design

The Dutch Birth Centre Study consists of five sub studies which are linked to one another:

1. Inventory of birth centres, development of quality indicators, definition of Birth Centre, measuring integration of organization and care
2. Measuring the quality of birth centre care
3. Effects of introducing a birth centre on regional quality and provision of care
4. Cost-effectiveness analysis
5. In depth longitudinal analysis of the organization and processes in birth centres

Different qualitative and quantitative methods will be used in the sub studies. Data collection includes observations, interviews (individual and group interviews), questionnaires (clients, caregivers, managers of birth centres), standard registered data and additional registrations. Data collected in one sub study will be shared with other sub studies as much as possible to make sure that the birth centres and other professionals involved in birth and postpartum care are minimally burdened by participating in the various evaluations. This study will be conducted in the period 2013–2015.

### Instruments

#### Dutch Birth Centre questionnaire

To characterize all Dutch birth centres the Dutch Birth Centre questionnaire will be developed based on the questionnaire of Laws et al. to characterize Australian birth centres [13]. This questionnaire includes questions about background, organisation and service of the birth setting: location, size, personnel, equipment, vision, management, judicial status, financial status, use of protocols, inter-professional cooperation and level of integration on six different domains (see below: measuring integration of organisation and care). It shall be adjusted to the Dutch situation with questions about transfer in case of referral, reasons for an obstetrician to come to the birth centre in case of urgent referral, facilities, postpartum stay, responsibility of care and potential quality indicators. Because quality indicators for birth centre care in the Netherlands are not available, they will be developed (see 1.2: developing quality indicators).

#### Repro-Q

Client-experiences will be assessed by using the postnatal part of the Repro-Q [14]. The Repro-Q consists of the following components: 1) characteristics of the process of care; 2) questions about the 8 domains of the concept of responsiveness of the World Health Organisation (WHO); 3) additional questions including experienced outcomes; 4) the valuation of the relative importance of the various domains; 5) the respondent’s socio-demographic characteristics [15].

#### Case report form

Individual baseline and outcome data are collected from the Netherlands Perinatal Registry (http://www.perinatreg.nl). The Netherlands Perinatal Registry (PRN-foundation) is a joint effort of the four professional organisations that provide perinatal care in the Netherlands: KNOV (Royal Organisation of Midwives in the Netherlands), LHV (National Organisation of General Practitioners), NVOG (Dutch Association of Obstetrics & Gynaecology) and NvK (Paediatric Association of the Netherlands). All professional organisations have their own voluntary based medical registry. Those registries are linked to one combined PRN-registry. The participation rate of obstetric caregivers (gynaecologists and midwives) is almost 100 %. All Dutch paediatricians working in a hospital with a neonatal intensive care unit (NICU) participate, as well as 60 % of the paediatricians working in hospitals without NICU [1].

To collect all additional process indicators and volumes for the different parts of the study a case record form shall be developed that includes (if applicable) date, time of day and dilatation at first and last visit at home before the actual birth and referral. We will also collect the time of start of continuous support by midwife and birth attendant, transport and arrival at birth centre or hospital, time of first action by secondary caregiver, time of arrival in birth centre postpartum and of the return home postpartum and number of hours of maternity care assistance at home. Furthermore data are collected about place of referral, type of transport in case of referral, discipline of the birth attendant and if the situation occurred that the preferred hospital or birth centre was fully booked.

### Outcome measures

Serious adverse outcomes are expected to be very low as the study population consists of women with an uncomplicated pregnancy who will start labour under midwifery-led care. Therefore the two main outcome measures will be composite measures: the Optimality Index (OI) and a composite measure of adverse neonatal and maternal outcomes [16].

The Optimality Index is a composite score combining background and outcome data based on a simple scoring system: optimal or not optimal. The optimal score is maximal perinatal outcome with minimal intervention placed against the woman’s health status. The OI is very suitable to compare groups with comparable risk profile or to correct group comparisons for differences in risk profile [17, 18]. Background data include age, parity, obstetric history, postal codes to characterize neighbourhood effects and social economic status, origin (Dutch or non-Dutch), together indicating the risk profile [19]. Elements included in the outcome part of the Optimality Index are for example: colour of amniotic fluid, induction/augmentation of labour, episiotomy, instrumental (vaginal) birth, Caesarean section, placental retention (>30 min) and Apgar score at 5 min.

The composite adverse outcome score will include maternal and neonatal outcome indicators. Adverse maternal outcome indicators are maternal death (within 42 days of giving birth), third or fourth degree of perineal trauma, placental retention, postpartum haemorrhage (>1000 ml), and admission to an intensive care unit or obstetric high care unit. Adverse neonatal outcome indicators are stillbirth after presentation in labour, early neonatal death (<7 days), Apgar score <7 after 5 min, neonatal encephalopathy, meconium aspiration, admission to neonatal unit within 48 h of birth and birth weight below 5th percentile.

### Description of sub studies

#### Sub study 1

##### Identification of birth centres, development of quality indicators, definition of Birth Centre, measuring integration of organization and care

The aim is to study the way birth centres are organised, what services are provided, who is responsible, and to measure the level of integration of care of birth centres.

### Identification of birth centres

Birth centres in their current presentation are relatively new in the Netherlands. Therefore no clear definition and no list of birth locations that can be considered a birth centre is currently available. To examine the large variety of possible birth centres criteria for inclusion are selected: birth settings where out-of-home community midwifery led care is provided in a home-like environment to women at low risk of medical complications at the onset of labour. Every birth location that can be included will be invited to participate in this part of the study. Based on the characteristics birth centres are examined by three independent researchers and a selection of all potential Dutch birth centres is made.

- Inclusion criteria participants: all locations in the Netherlands that could be considered a birth centre
- Method: systematic inquiries
- Expected outcome: identification of all potential Dutch birth centres (reference date August 2013)

### Developing a comprehensive set of structure and process quality indicators for birth centre care

A comprehensive set of structure and process quality indicators will be developed to evaluate birth centre care using a multi-staged approach. The development process consists of three phases: 1) identification of existing structure and process quality indicators in birth care (literature study); 2) translating indicators for maternity care in general into determinants for measuring structure and process quality of birth centre care; 3) determinant selection of relevant structure and process quality indicators (two-step web-based Delphi consultation) [20]. The web-based, anonymous nature of the Delphi technique ensures that a single individual cannot dominate the consensus formation. Professionals from different disciplines who are working with or in a birth-centre-like setting with several years of experience, representatives of health insurance companies, policymakers and advisors will be invited to participate in the Delphi consultation. The experts are instructed to rate the determinants both on relevance to a birth centre setting and on feasibility of use and, if necessary, to comment on them or add new topics. Each determinant will be rated by each expert on a seven-point Likert scale (1 = not at all relevant/feasible; 4 = neutral; 7 = very much relevant/feasible). Agreement among experts is defined as 80 % or more of the ratings within a range of three (i.e. 5-6-7 of 4-5-6). In the first round determinants with a median score of ≥ 6 with agreement on both ratings are considered to be relevant and feasible to collect and are accepted instantly. Determinants scored with a median score of ≤3 are rejected. Median scores of >3 and <6 with agreement or ≥6 without agreement are scored again in the second Delphi round. In the second round, the experts are informed about the median scores of relevance and feasibility of the total expert group, their own scores and the comments of the respondents regarding determinants for which no consensus is reached in the first round. They are instructed to re-consider their own rating of the determinants presented in the first round as well as to rate and comment possible new elements the same way as in the first round.

This procedure will result in a list of potential structure and process quality indicators for birth centres in the Netherlands. In order to test whether these quality indicators actually can measure the quality of birth centres, they will be validated within the presumed selection of birth centres.

- Inclusion criteria participants: professionals working with or in a birth centre, representatives of health insurance companies, policymakers and advisors
- Method: two-step web-based Delphi consultation
- Instrument: web-based questionnaire
- Expected outcome: a list of potential structure and process quality indicators for birth centres in the Netherlands

### Definition of a birth centre in the Netherlands

The Dutch Birth Centre questionnaire will be sent to a (management) representative of each birth location as identified in the first step of the study. A definition for different types of birth centres in the Netherlands will be developed based on internationally used definitions and the information obtained through our questionnaire. The characteristics of all Dutch birth centres will be described.

- Inclusion criteria participants: management representatives in all birth locations identified previously
- Method: survey
- Instrument: Dutch Birth Centre questionnaire (adjusted Laws questionnaire)
- Expected outcome: preliminary classification/typology of birth locations into birth centres and other birth settings

### Measuring integration of organization and care

To construct a typology of birth centres we will use the concept of integrated care. This concept was developed first for the increasing number of people with a chronic disease. Different (health-related) disciplines are involved in the continuous care for persons with a chronic disease. For instance, care for a person with diabetes mellitus type II may involve a general practitioner, a dietician, and a physiotherapist, but also an endocrinologist. The essence of integrated care is a continuum of care for service users which crosses the boundaries of primary, secondary, tertiary and public health care [20–22]. The definition of the WHO illustrates the extensive conceptualization of integrated care: “a concept bringing together inputs, delivery, management and organization of services related to diagnosis, treatment, care, rehabilitation and health promotion. Integration is a means to improve the services in relation to access, quality, user satisfaction and efficiency” [23]. Domains of integration are 1) clinical, 2) professional, 3) organisational, 4) systemic, 5) functional, and 6) normative integration [24]. Based on the scores on the different domains an overall score of integration will be calculated to define the level of integration for each birth centre as low, medium or high.

- Inclusion criteria participants: all birth locations identified preliminary as birth centre
- Method: survey and interview
- Instruments: Dutch Birth Centre questionnaire, interview topic list, conceptual framework on integrated care
- Expected outcome: level of integration for each birth centre

#### Sub study 2

##### Measuring the quality of birth centre care

The aim is to study the process and outcomes of birth centre care, compared to birth at home and birth in a hospital, for pregnant women under the responsibility of the independently operating community midwife at the start of labour. Client experiences and provider satisfaction are included in the outcome measures. At the end of the total study all different outcomes will be linked with each other.

### Measuring process and outcomes of birth centre care

Midwifery practices in the area of all birth locations in this study will record the data for each birth under their care during 3 months: data routinely recorded in the Netherlands Perinatal Registry and additional process indicators not available from the Netherlands Perinatal Registry.

- Inclusion criteria participants: all low risk women starting labour while in care with a participating community midwife for a period of 3 months
- Method: standard and additional health care registration
- Instruments: Optimality Index and a composite measure of adverse neonatal and maternal outcome
- Expected outcome: quality of care in birth centres versus home or hospital birth for low risk women

### Client experiences

To assess client experiences the postnatal part of the Repro-Q will be used. Especially for this study, questions about facilities and transfer are added for women who received care in a birth centre. The same midwifery practices as in sub study 2.1 will be asked to distribute information of this part of the study and a acceptance paper form to each woman that receives care in their postpartum period regardless who gave natal care to them. These women will be approached 6 to 8 weeks after they give birth by the way they preferred to answer the questions on client experiences i.e. by email, by post or by telephone. A reminder will be sent after 4 weeks.

- Inclusion criteria participants: all women in their postpartum period under care of participating community midwives for a period of 3 months
- Method: Survey
- Instrument: Repro-Q with added questions
- Expected outcome: women’s experiences with perinatal care

### Care providers experiences

To assess the experiences of professionals working within and with a birth centre a questionnaire will be developed based on earlier questionnaires used in workforce planning [25, 26]. The development will be a joint effort with other Dutch researchers to create a multipurpose questionnaire. The questionnaire will contain questions about personal background, current job situation, cooperation with other care providers, current job evaluation and future job situation and will be sent to all care givers working in or with a birth centre like community midwives, clinical midwives, obstetricians, paediatricians and maternity care assistants.

- Inclusion criteria participants: all care providers working in and with birth centres in the Netherlands
- Method: Survey
- Instruments: Care provider questionnaire
- Expected outcome: providers’ experiences and satisfaction

#### Sub study 3

##### Effects of introducing a birth centre on regional quality and provision of care

The aim of the evaluation is to gain insight into the effect of the introduction of a birth centre in a region on planned place of birth and the outcomes of the provided birth and postpartum care.

### Process and outcome

In May 2011 a baseline assessment was performed in areas where a birth centre was intended to start before June 2013. Ten regions collected data for more than 3 months. Midwifery practices in the area of an intended birth centre recorded the following data for each birth under their care: data routinely recorded in the Netherlands Perinatal Registry and additional process indicators not available from the Netherlands Perinatal Registry (see sub study 2). The follow-up measurement has been conducted in the second half of 2013. Data collection has resulted in around 3000 births for the pre-test period and will result in 3000 births for the post-test period. These numbers are sufficient to describe changes in the region between the period before the birth centre started and afterwards. Logistic regression analysis will be performed to study the difference in planned place of birth between the period before and after the start of the birth centre. Linear regression analysis will be performed to test the mean differences in the Optimality Index between the period before and after the birth centre started. All analyses will be adjusted for potential confounders such as maternal age, parity and gestational age.

- Inclusion criteria participants: all low risk women starting labour while in care with a participating community midwife for a period of at least 3 months before the start of the birth centre and a minimum of 3 months afterwards
- Method: standard and additional health care registration
- Instruments: case record form, Optimality Index and a composite measure of adverse neonatal and maternal outcome
- Expected outcome: effect of the start of a birth centre on regional quality of care for low risk women

#### Sub study 4

##### Cost-effectiveness analysis

The costs and effects of women with planned place of birth at a birth centre will be compared to women with a planned place of birth home and hospital under midwifery led care.

### Effects

The outcome measure for the effect study will be the Optimality Index. At least three midwifery practices in the area of each birth centre in this study will record data for each birth that started under their care during 3 months: data routinely recorded in the Netherlands Perinatal Registry and additional process indicators not available from the Netherlands Perinatal Registry (see sub study 2). A sample size of nine birth centres per level of integration (low, medium, high, see sub study 1.4) with 66 women per centre achieves 80 % power to detect an effect size of 0.2 (ICC = 0.005, alpha = 0.05).

- Inclusion criteria participants: all low risk women starting labour while in care with a participating community midwife and living and having a birth centre as an option for planned place of birth
- Method: standard and additional health care registration
- Instruments: case record form, Optimality Index and a composite measure of adverse neonatal and maternal outcome
- Expected outcome: effect of planned place of birth (home, conventional labour setting or birth centre) on regional quality of care for low risk women

### Costs

For the births included in the effectiveness part the costs will be assessed. Costs of birth in this study include the health care costs from the start of labour until 7 days after birth. These costs consist of a) medical interventions during birth such as: referral, augmentation, pharmaceutical and non-pharmaceutical pain relief, continuous foetal monitoring, intra partum antibiotics prophylaxis, continuous support of labour, birth by caesarean section, instrumental vaginal birth, manual placenta removal and blood transfusion, b) use of hospital facilities such as: hospital admission and length of stay, and c) staffing such as: attending midwife or obstetrician or both, maternity care assistance during childbirth and in the days thereafter.

Volume of health care resource use will be registered prospectively on the case record form used by the attending midwife. Costs of birth and postpartum care are estimated by a detailed cost price analysis. Other resource use (e.g. hospital days) will be translated into costs using standard prices [25].

Total costs per woman according to planned place of birth will be calculated. Mean differences between the groups and their 95 % confidence intervals will be estimated using non parametric bootstrapping due to the skewed nature of cost data.

- Inclusion criteria participants: all low risk women starting labour while in care with a participating community midwife and living and having a birth centre as an option for planned place of birth for a period of at least 3 months
- Method: measurement of quantities and assignment of unit costs by detailed cost price analysis and use of standard prices
- Instruments: case record forms
- Expected outcome: effect of planned place of birth on costs

### Economic evaluation

The aim of the economic evaluation is to study the cost-effectiveness of the care provided by different types of birth centres compared to home birth and hospital birth under midwifery led care. The economic evaluation will be performed from a health care perspective. The time horizon of the economic evaluation is from the start of labour until 7 days after birth. Due to this short time frame no discounting will take place.

Costs and effects (as measured by the Optimality Index) will be transformed in a net-monetary benefit (NMB) estimate. Using the net benefit regression approach cost-effectiveness acceptability curves will be generated which show the probability of being cost-effective for the different planned places of birth: at home, the different types of birth centres and hospital birth for all acceptable levels of the willingness to pay [27].

- Inclusion criteria participants: all women starting labour while in care with a participating community midwife during a period of 3 months
- Method: incremental net benefit method
- Instruments: case record form and Optimality Index
- Expected outcome: cost-effectiveness of birth centre care compared to home or hospital birth for low risk women

#### Sub study 5

##### In depth longitudinal analysis of the organization and processes in birth centres

The aim of this study is to assess to what extent different degrees of organizational integration (on the continuum from partial to fully integrated obstetric care) lead to differences in performance.

### Design and longitudinal in depth analysis

This longitudinal qualitative research focuses on arriving at a deeper level of understanding of the process of care and cooperation and its development over time. The research design for this study is that of a process study using the grounded theory methodology [28]. Seven birth centres will be selected after an initial first exploratory round of visits by theory-driven case selection [29].

Data will be collected through investigating from a so-called engaged scholarship/quasi-ethnographic perspective, in which from a variety of data sources over a substantial period of time conclusions will be drawn. This means that observations will take place in each of the birth centres for a number of days at a time, during daytime and during night-time, to observe ongoing activities and to interview care providers as well as clients.

Data will be analysed using the constant comparative method. The purpose is to attain new insights by breaking through standard ways of thinking about phenomena reflected in the data [28]. In this way concepts emerge as theory is formed. Analysis will start as soon as the first data are collected and continue with each additional data collection. The first step in the analysis will be coding the transcripts of the observations and interviews. The analysis and findings will be based on a triangulation of different types of data [30]. First, the researcher will make comprehensive detailed field notes of the observations and informal conversations. Second, surveys will be used. Third, qualitative dimensions such as distances between birth centre and obstetrical ward and time needed for transfer in case of referral will be measured. Fourth, a member-check will be conducted to verify the collected information. Fifth, the researcher will keep a diary in which she reports her own behaviour and feelings, as distinct from her observations in the field notes. Sixth, peer-reviewing will be conducted by evaluation of the work by one or more colleagues.

- Inclusion criteria participants: birth centres selected by theory-drive selection
- Method: observations and interviews
- Instruments: fly-on-the-wall observations, topic list for interviews, member checks and peer reviews
- Expected outcome: improved understanding how different aspects of organizational design, care processes and collaboration (a) interrelate and (b) how they affect (non-medical) outcomes

### Overall analysis

The insights of all sub studies will be put together, whereby the various elements of the research will be integrated. The national quantitative results will be combined with the insights from the interviews, the cost effectiveness results, client and professional experiences and the information and mirror sessions of the in-depth study to provide insight in the quality of birth centre care in the Netherlands. The regional quantitative results will provide insight into the development over time in a changing health care setting. Based on the confrontation of the various kinds of information more insight can be gained about birth centre care in general and about the strengths and weaknesses of different ways to organise birth centres in particular.

This form of triangulation of information that results from various scientific paradigms is an exciting process that will be carried out by the principal investigators of the participating organizations. It will lead to recommendations for further development of birth centres in the Netherlands.

### Considerations

This study will be carried out by an unique collaboration of several organizations, each with their own proven expertise in the field of the organization of health care and perinatal care in particular. Prior to as well as during the study period all organizations will be involved in both the planning and execution of all related sub studies. A broad advisory committee will be formed by representatives of all different kind of maternity care providers, research consortia, professional organizations, health insurance companies, national health department and clients to discuss the process and preliminary outcomes of this study. Design and planning of the study were presented to the Medical Ethics Committee of the UMCU (University Medical Centre Utrecht). They confirmed that this study agrees with Dutch legal regulations for the methods used for this study and because of that official ethical approval of this study is not required [31]. 

## Discussion

The Dutch Birth Centre study will evaluate the effect of birth centre care in the Netherlands from different angles and combining different research methods. In this way the Dutch Birth Centre Study will provide information on the functioning of different birth centres as well as their contribution to the quality of birth and postpartum care and the effect of the level of integration on the organisation of birth centre care; it also will evaluate the quality of birth centre care in terms of process and health outcomes, compared to birth at home or on a maternity ward in a hospital. Client and provider experiences are included in the outcome measures. An economic evaluation will assess cost-effectiveness of birth centre care compared to care as usual (i.e. home birth and hospital birth). In-depth analysis will provide information on how different degrees of organizational integration on the continuum from partial to fully integrated birth care will lead to differences in performance.

In 2009 a steering committee instituted by the Dutch ministry of Health published a report called ‘A good start’ (in Dutch: ‘Een goed begin’) [32]. This offered Dutch maternity care givers tools to help to improve their performance and because of that perhaps lower the relatively high mortality rates in the Netherlands [33]. This report also pointed out that birth centres might play a role in improving perinatal outcome but only if the surplus value could be demonstrated. This study aims to evaluate the performance of birth centres and their possible added value to the Dutch maternity care system.

The sudden increase in birth centres as integral part of the maternity care system is a relatively new development in the Netherlands. Until now, it seems that each region is developing its own version, based on local preferences, available space, and (lack of) mutual trust. Generally applicable standards for birth centre care are not available and there is no evidence of their added value. This study is designed to fill these gaps in our knowledge, to provide minimum standards for birth centre care and to compare their performance to the traditional care provision at home or in a hospital.

The results of this study will enable care providers, policy makers, health care financers, professionals and users of maternity care to make an educated choice about the kind of birth location that is appropriate for their needs and wishes.

## Abbreviations

KNOV: Royal Organisation of Midwives in the Netherlands
LHV: National Organisation of General Practitioners
NICU: Neonatal intensive care unit
NMB: Net-monetary benefit
NvK: Paediatric Association of the Netherlands
NVOG: Dutch Association of Obstetrics & Gynaecology
OI: Optimality Index
PRN: The Netherlands Perinatal Registry
WHO: World Health Organisation
ZonMW: Netherlands Organisation for Health Research and Development
